# The Na^+^/H^+^ exchanger contributes to increased smooth muscle proliferation and migration in a rat model of pulmonary arterial hypertension

**DOI:** 10.14814/phy2.12729

**Published:** 2016-03-20

**Authors:** John C. Huetsch, Haiyang Jiang, Carolina Larrain, Larissa A. Shimoda

**Affiliations:** ^1^ Division of Pulmonary and Critical Care MedicineDepartment of MedicineJohns Hopkins School of MedicineBaltimoreMaryland

**Keywords:** Intracellular pH, migration, proliferation, pulmonary hypertension, smooth muscle, sodium–hydrogen exchanger

## Abstract

Increased muscularity of small pulmonary vessels, involving enhanced proliferation and migration of pulmonary arterial smooth muscle cells (PASMCs), is a key component of the vascular remodeling underlying the development of pulmonary hypertension (PH). Stimuli such as growth factors and hypoxia induce PASMC alkalinization, proliferation, and migration through upregulation of the Na^+^/H^+^ exchanger (NHE), inhibition of which prevents the development of hypoxia‐induced vascular remodeling and PH. We wanted to explore whether NHE was also necessary for pathologic PASMC proliferation and migration in a model of pulmonary arterial hypertension (PAH), a severe form of PH not associated with persistent hypoxia. PASMCs were isolated from rats exposed to SU5416‐hypoxia (SuHx) followed by return to normoxia and from vehicle controls. We measured resting intracellular pH (pH_i_) and NHE activity using the pH‐sensitive fluorescent dye BCECF‐AM. PASMC proliferation and migration were assessed using BrdU incorporation and transwell filters, respectively. NHE activity was increased in SuHx PASMCs, although resting pH_i_ was unchanged. SuHx PASMCs also exhibited increased proliferation and migration relative to controls, which was attenuated in the setting of pharmacologic inhibition of NHE. Our findings suggest that increased NHE activity contributes to pathologic PASMC function in the SuHx model of PAH, although this effect does not appear to be mediated by global changes in pH_i_ homeostasis.

## Introduction

Pulmonary hypertension (PH) is classified into five etiologic groups. Group 1 PH, known as pulmonary arterial hypertension (PAH), is the subset of PH characterized by pathology of the precapillary vasculature, while Group 3 PH is the subset due to underlying lung disease or chronic hypoxia (CH) (Simonneau et al. 2009). In PAH, elevation of pulmonary arterial pressure is due to a combination of sustained vasoconstriction and structural remodeling (Oka et al. 2007; Tuder 2009; Shimoda and Laurie 2013). Significant components of remodeling in PAH include medial thickening in muscularized pulmonary vessels as well as extension of muscularization distally to small pulmonary arteries (Chazova et al. 1995; Tuder 2009), both of which are associated with enhanced proliferation (Falcetti et al. 2010) and migration (Paulin et al. 2014) of pulmonary arterial smooth muscle cells (PASMCs). The continued high mortality of PAH, despite the advent of several classes of primarily vasodilatory therapies, highlights the importance of understanding mechanisms underlying vascular remodeling in the pursuit of new therapies (Ling et al. 2012).

Although the cellular changes that underlie the alterations in PASMC function during PAH remain incompletely understood, modulation of intracellular pH (pH_i_) is one mechanism known to regulate both proliferation (Quinn et al. 1996; Wu et al. 2006; Yu and Hales 2011) and migration (Wu et al. 2006; Yu and Hales 2011) of PASMCs. pH_i_ homeostasis in mammalian cells is maintained by a combination of ion exchange mechanisms: Na^+^/HCO_3_^−^ cotransport, Na^+^‐dependent Cl^−^/HCO_3_^−^ exchange, Na^+^‐independent Cl^−^/HCO_3_^−^ exchange, and Na^+^/H^+^ exchange (NHE), with all but Na^+^/HCO_3_^−^ cotransport known to be active in PASMCs (Huetsch and Shimoda 2015). NHE, in particular, has been shown to play an important role in regulation of resting pH_i_ and recovery from acid load in PASMCs from a spectrum of organisms (Quinn et al. 1991, 1996; Farrukh et al. 1996; Madden et al. 2001; Rios et al. 2005; Shimoda et al. 2006). NHEs are a family of transmembrane ion exchangers which alkalinize the cell through export of H^+^ and import of Na^+^. The NHE family encompasses at least 10 known isoforms, with NHE1 ubiquitously expressed and other isoforms localized to specific tissues. NHE1 and NHE2 are expressed in whole lung tissue preparations (Orlowski et al. 1992; Wang et al. 1993), while NHE3–5 are not (Orlowski et al. 1992; Brant et al. 1995; Attaphitaya et al. 1999). The localization of NHE6–10 is known to be restricted to limited tissue types or to intracellular organelles (Numata et al. 1998; Miyazaki et al. 2001; Numata and Orlowski 2001; Nakamura et al. 2005). NHE1, but neither NHE2 nor NHE3, was found to be expressed in mouse (Rios et al. 2005) and rat (Shimoda et al. 2006) PASMCs, suggesting NHE1 as the primary isoform responsible for PASMC cytosolic pH_i_ homeostasis.

Stimuli such as growth factors (i.e., platelet‐derived growth factor and epidermal growth factor) (Quinn et al. 1996) and CH (Rios et al. 2005) induce PASMC alkalinization, which is mediated by increased NHE activity. NHE activity is important for stimulated PASMC function, as pharmacologic inhibition of NHE attenuated PASMC proliferation in response to growth factors (Quinn et al. 1996) and specific silencing of NHE1 blunted enhanced proliferation and migration of human PASMCs in response to hypoxia (Yu and Hales 2011). Furthermore, NHE1 was necessary for the development of PH in response to CH, as either pharmacologic (Quinn et al. 1998) or genetic (Yu et al. 2008) inhibition of NHE1 prevented CH‐induced vascular remodeling and PH. NHE1 mRNA and protein expression were increased in PASMCs isolated from chronically hypoxic rodents as well as in PASMCs exposed to hypoxia ex vivo after isolation from normoxic rodents, indicating that upregulation of NHE is a direct effect of hypoxia (Shimoda et al. 2006). Thus, while NHE is clearly important to pathologic PASMC function and subsequent development of PH in response to hypoxia, it is unknown whether NHE is similarly relevant to the pathogenesis of other classes of PH that are not associated with persistent hypoxia, such as PAH.

In contrast to CH animal models of PH, the SU5416‐hypoxia (SuHx) rat model, which consists of treatment with an antagonist of vascular endothelial growth factor receptor combined with exposure to hypoxia followed by return to normoxia, recapitulates key components of human PAH, including severely elevated pulmonary arterial pressures, development of vaso‐occlusive lesions, and persistence of robust vascular remodeling following return to normoxia (Taraseviciene‐Stewart et al. 2001; Abe et al. 2010). Indeed, whereas the pulmonary vasculature in CH models begins to de‐remodel upon return to normoxic conditions, remodeling in the SuHx model continues to progress even after cessation of hypoxic exposure. However, whether PASMCs isolated from this model exhibit alterations in pH_i_ homeostasis, NHE activity, or proliferation and migration remains unknown. Thus, in this study, we assessed NHE activity and its role in PASMC proliferation and migration in the SuHx rat model of PAH.

## Methods

### SuHx exposure

All procedures were approved by the Animal Care and Use Committee of The Johns Hopkins University School of Medicine. Male Wistar rats were injected with SU5416 (20 mg/kg s.c.) on day 1, placed in a hypoxic chamber from days 1 to 21, and then returned to normoxia on days 21 to 35. SU5416 was suspended in a mixture of dimethyl sulfoxide (DMSO) and CMC (0.5% [w/v] carboxymethylcellulose sodium, 0.9% [w/v] sodium chloride, 0.4% [v/v] polysorbate 80, 0.9% [v/v] benzyl alcohol in deionized water). The hypoxic chamber was continuously flushed with a mixture of room air and N_2_ (10 ± 0.5% O_2_) to maintain low CO_2_ concentrations (<0.5%). Chamber O_2_ and CO_2_ concentrations were continuously monitored (ProOx 110 oxygen analyzer; Biospherix, Redfield, NY, and LB‐2 CO_2_ analyzer; Sensormedics, Anaheim, CA). The rats were exposed to room air for 10 min twice a week to clean the cages and replenish food and water supplies. Normoxic controls were injected s.c. with SU5416 vehicle on day 1 and kept in room air next to the hypoxic chamber from days 1 to 35. Thus, all animals were exposed to the same light/dark cycle and ambient temperatures. On day 35, rats were anesthetized with pentobarbital sodium (43 mg/kg ip) and sacrificed.

### Right ventricle (RV) pressure measurement

Closed‐chest RV pressure was measured in anesthetized rats through an abdominal incision, as previously described in mice (Yu et al. 1999). The diaphragm was visualized through the abdomen and RV pressure was measured via a 23‐gauge needle filled with heparinized saline and connected to a pressure transducer (model P10EZ; Spectramed, Oxnard, CA). Correct localization of the puncture was verified by postmortem inspection. Pressure was recorded using Power Lab Software (ADI Instruments, Colorado Springs, CO) and a sampling rate of 200 per sec. Only rats in which stable tracings were obtained and RV puncture was verified were included in the analysis. RV systolic pressure (RVSP) was determined as the average peak pressure from a minimum of five heartbeats.

### Hematocrit (Hct) measurement

Blood was collected from the left ventricle (LV) via a 23‐gauge needle attached to a syringe coated with EGTA and placed in EGTA‐treated tubes. The blood was mixed and a small amount drawn into a capillary tube, the plasma separated via low‐speed centrifugation, and Hct measured using a microHct capillary tube reader chart.

### RV hypertrophy (RVH) determination

After RV pressure was measured, the heart and lungs were removed en bloc and transferred to a Petri dish of HEPES‐buffered salt solution (HBSS) containing (in mmol/L) 130 NaCl, 5 KCl, 1.2 MgCl_2_, 1.5 CaCl_2_, 10 HEPES, and 10 glucose, with pH adjusted to 7.2 with 5 mol/L NaOH. Under a dissecting microscope, the atria and extraneous vascular material were removed from the heart. The RV wall was carefully separated from the left ventricle and the septum (LV+S), and both portions were blotted dry and weighed.

### Lung histology

In a subset of animals, sutures were used to occlude the right lower lobe and left upper lobe, which were removed for isolation of PASMCs. A cannula was inserted into the trachea, and the remaining lobes were inflated and fixed with 1.5 mL of 10% formalin, embedded in paraffin, and sectioned into 5 *μ*m slices. Sections were subjected to antigen retrieval, blocked, and either briefly stained with hematoxylin and eosin or exposed to smooth muscle‐specific *α*‐actin (SMA) antibody (Sigma Aldrich, St. Louis, MO) overnight at 4°C, and incubated with peroxidase‐labeled secondary antibody (KPL, Gaithersburg, MD) for 1 h at room temperature, following by staining with addition of 3,3′‐diaminobenzidine (DAB). For each lung, 20 high‐powered fields (hpfs) were randomly selected and photographed (Nikon TMS‐F microscope, Melville, NY). Vascular hypertrophy was measured as % wall thickness, calculated by ([diameter_ext_ – diameter_int_]/diameter_ext_)/2 × 100. Dimensions were demarcated by the external (ext) and internal (int) elastic laminae and measurements were taken in ImageJ, examining >20 vessels (all <250 *μ*m in diameter) each from SuHx and control rats. Vaso‐occlusive lesions were tallied in 20 randomly selected and photographed hpfs from each rat by an investigator blinded to exposure condition.

### Isolation of PASMCs

Intralobar pulmonary arteries (200–600 *μ*m outer diameter) were dissected and cleaned of connective tissue in ice‐cold HBSS as previously described (Rios et al. 2005). The arteries were opened and the lumen gently rubbed to remove the endothelium. Cleaned arteries were allowed to recover for 30 min in cold (4°C) HBSS followed by 20 min in reduced Ca^2+^ HBSS (20 *μ*mol/L CaCl_2_) at room temperature. After recovery, the tissue was incubated for 15 min at 37°C in reduced Ca^2+^ HBSS containing collagenase (type I; 1750 U/mL, Worthington, Lakewood, NJ), papain (12.35 U/mL, Sigma, St. Louis, MO), bovine serum albumin (2 mg/mL), and dithiothreitol (1 mmol/L). Single SMCs were dispersed by gentle trituration of the tissue in Ca^2+^‐free HBSS. Cells were expanded in SmGM complete media (Lonza, Basel, Switzerland) supplemented with 1% penicillin/streptomycin and then placed in basal media (SmBM plus 0.3% fetal calf serum [FCS] and 1% penicillin/streptomycin) 24 h before experiments.

### Immunofluorescence

PASMCs grown on glass slides were washed, fixed, and permeabilized. Cells were then incubated with antibodies against myosin heavy chain 11 (MHC, Abcam, Cambridge, UK) or calponin (Abcam) followed by incubation with fluorescent‐conjugated secondary antibody (Cy3 goat anti‐mouse IgG, Life Technologies, Carlsbad, CA) plus DAPI nuclear counterstain, and then observed using a microscope with fluorescence objectives (Olympus IX51, Center Valley, PA). The percent of cells which were verified as PASMCs was measured as the # of cells positive for PASMC marker/the # of cells positive for DAPI.

### pH_i_ measurements

PASMCs were placed in a laminar flow cell chamber perfused with either Krebs bicarbonate‐buffered physiologic salt solution (PSS) containing (in mmol/L): 118 NaCl, 4.7 KCl, 0.57 MgSO_4_, 1.18 KH_2_PO_4_, 25 NaHCO_3_, 2.5 CaCl_2_, and 10 glucose gassed with 16% O_2_–5% CO_2_, or a HEPES‐buffered PSS containing (in mmol/L): 130 NaCl, 5 KCl, 1 MgCl_2_, 1.5 CaCl_2_, 10 glucose, and 20 HEPES with pH adjusted to 7.4 with NaOH (HEPES1 solution). pH_i_ was measured in cells incubated with a membrane‐permeant (acetoxymethyl ester) form of the pH‐sensitive fluorescent dye BCECF (Life Technologies) for 60 min at 37°C under an atmosphere of 21% O_2_–5% CO_2_. Cells were then washed with PSS for 15 min at 37°C to remove extracellular dye and allow complete de‐esterification of cytosolic dye. Ratiometric measurement of fluorescence from the dye was performed on a workstation (Intracellular Imaging, Cincinnati, OH) consisting of a Nikon TSE 100 Eclipse inverted microscope with epifluorescence attachments. The light beam from a xenon arc lamp was filtered by interference filters at 490 and 440 nm and focused onto the PASMCs under examination via a 20X fluorescence objective (Super Fluor 20, Nikon, Melville, NY). Light emitted from the cell at 530 nm was returned through the objective and detected by an imaging camera. An electronic shutter (Sutter Instruments, Novato, CA) was used to minimize photobleaching of dye. Protocols were executed and data were collected online with InCyte software (Intracellular Imaging). pH_i_ was estimated from in situ calibration after each experiment. Cells were perfused with a solution containing (in mmol/L): 105 KCl, 1 MgCl_2_, 1.5 CaCl_2_, 10 glucose, 20 HEPES, and 0.01 nigericin to allow pH_i_ to equilibrate to external pH. A two‐point calibration was created from fluorescence measured as pH_i_ was adjusted with KOH from 6.1 to 7.5.

For resting pH_i_ measurements, PASMCs loaded with BCECF were placed on the fluorescence microscope and perfused at a rate of 0.75 mL/min in PSS (either Krebs or HEPES1) for 3 min and then in PSS containing 10 *μ*mol/L ethyl‐isopropyl amiloride (EIPA) for 15 min.

A standard ammonium pulse technique was used to measure NHE activity in bicarbonate‐free PSS (Huetsch and Shimoda 2015). PASMCs loaded with BCECF were placed on the fluorescence microscope and perfused at a rate of 0.75 mL/min with HEPES1 solution for 2 min. PASMCs were then briefly exposed to NH_4_Cl (ammonium pulse) by perfusing with HEPES2 solution containing (in mmol/L): 110 NaCl, 20 NH_4_Cl, 5 KCl, 1 MgCl_2_, 1.5 CaCl_2_, 10 glucose, and 20 HEPES at a pH of 7.4 using NaOH for 3 min. The ammonium pulse caused alkalinization due to influx of NH_3_ and buffering of intracellular H^+^. Washout of NH_4_Cl in the absence of extracellular Na^+^ using a Na^+^‐ and NH_4_^+^‐free solution (HEPES3) containing (in mmol/L): 130 choline chloride, 5 KCl, 1 MgCl_2_, 1.5 CaCl_2_, 10 glucose, and 20 HEPES at a pH of 7.4 using KOH for 10 min resulted in acidification due to rapid diffusion and washout of NH_3_, leaving behind H^+^ ions. The external solution was then switched back to HEPES1 solution for 10 min. Readdition of extracellular Na^+^ allowed activation of NHE and recovery from acidification to basal levels. NHE activity was measured as the rate of Na^+^‐dependent recovery from intracellular acidification in the first 2 min after re‐exposure to HEPES1. To measure the effect of EIPA upon NHE activity, PASMCs were incubated with 10 *μ*mol/L EIPA for 60 min prior to ammonium pulse protocol, which then included 10 *μ*mol/L EIPA in the HEPES1, 2, and 3 solutions. To measure the effect of rho kinase (ROCK) inhibition upon NHE activity, PASMCs were incubated with 10 *μ*mol/L Y‐27632 (Abcam) for 60 min prior to ammonium pulse protocol, which included 10 *μ*mol/L Y‐27632 in the HEPES1, 2, and 3 solutions.

### Immunoblot assay

Endothelium‐denuded intralobar pulmonary arteries dissected from rats were flash frozen in liquid nitrogen. The vessels were placed in T‐PER (ThermoFisher Scientific, Waltham, MA) and sonicated (BioLogics Ultrasonic Homogenizer Model 150VT, Manassas, VA) to extract PASMC protein. Protein concentrations were calculated from a standard BCA assay. For each sample, protein was separated on 8% SDS‐PAGE gels and transferred to nitrocellulose membranes. Membranes were blocked in 5% nonfat milk for 1 h and probed with anti‐NHE1 antibody (Millipore, Billerica, MA) overnight at 4°C, washed, and incubated in peroxidase‐labeled secondary antibody (KPL) for 1 h. Bands were visualized by enhanced chemiluminescence according to the manufacturer's instructions. Membranes were then probed for *β*‐tubulin, as a loading control. Densitometry was performed in ImageJ to quantify the amount of protein, and the ratio of NHE1 to *β*‐tubulin was calculated. Fold induction was determined by setting the ratio of NHE1/*β*‐tubulin in normoxic animals equal to 1.

### PASMC migration

Cell migration was assessed using transwell filters (Corning, NY). Cells (50,000) were added to the top of a polycarbonate filter with 8 *μ*m pores in 4 mL of basal media. Either 10 *μ*mol/L EIPA or vehicle (DMSO) was added to the media 1 h after cell seeding, and the cells were incubated for a subsequent 24 h. PASMCs were then fixed in 95% ethanol for 10 min, washed with PBS, and stained with brilliant blue (Sigma) for 5 min. Cells were visualized via a microscope‐mounted camera and Q‐capture software. For each filter, five randomly chosen fields were imaged to obtain a total adherent cell count. Unmigrated cells were then scraped off the top of the filter and the migrated cells (bottom layer) were imaged. Migration was calculated as a percentage (# of migrated cells/# of total cells).

### PASMC proliferation

Cell proliferation was determined using a commercially available kit for BrdU incorporation (GE Healthcare Life Sciences, Pittsburgh, PA). Each well was plated with 5000 cells in basal media. BrdU, in addition to either 10 *μ*mol/L EIPA or vehicle, was added to each well 1 h after cell seeding. BrdU incorporation was measured 24 h later.

### Data analysis

Data are expressed as means ± SE, *n* is the number of animals used in an experiment. For experiments in which pH_i_ was measured, data were collected from 10 to 30 cells per animal and averaged to obtain a single value for each animal. Statistical comparisons were performed using Student's *t* test or two‐way ANOVA, as appropriate. Pairing (repeated measures) was utilized in ANOVA analyses when PASMCs from an individual rat were subjected to both vehicle and drug. For ANOVA analyses, multiple comparisons testing was performed post hoc using the Sidak test. Differences were considered to be significant when *P < *0.05. All significant interactions from ANOVA analyses are noted in the results.

## Results

### Physiologic and hemodynamic measures in the SuHx model

**Figure 1:**
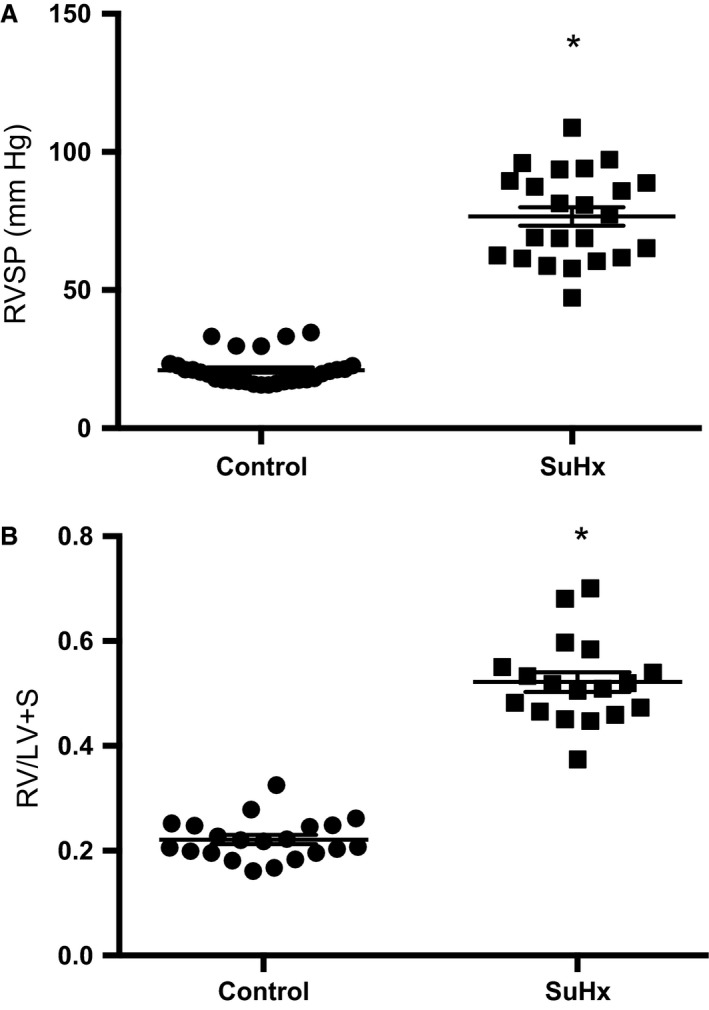
Physiologic effect of SU5416‐hypoxia (SuHx) exposure in rats. (A) Plot of right ventricular systolic pressure measured via transdiaphragmatic puncture in control (*n *=* *30) and SuHx (*n *=* *23) rats. (B) Plot of RVH measured as right ventricle/left ventricle + septum (RV/LV+S) weight in control (*n *=* *21) and SuHx (*n *=* *18) rats. Lines indicate means with standard errors. *indicates significant difference between control and SuHx values (*P *<* *0.05 by unpaired *t* test).

To verify the development of PH in rats exposed to SuHx, RVSP and RVH were measured. RVSP (Fig. 1A) was significantly elevated in the SuHx rats (76.65 ± 3.37 mmHg, *n* = 23) compared to normoxic controls (21.04 ± 1.02 mmHg, *n* = 30). RVH was also significantly elevated in the SuHx rats (Fig. 1B). When adjusted for body weight, RV weight was greatly increased in SuHx rats while LV+S weight was mildly increased (Table 1). Hct was not different in the SuHx group, indicating any polycythemia that may have occurred as a result of the hypoxic exposure had normalized with the 2‐week posthypoxic recovery in normoxic conditions. These results indicate that SuHx rats developed severe PH and are similar with past results (Taraseviciene‐Stewart et al. 2001; Oka et al. 2007).

**Table 1:** Effect of SU5416‐hypoxia (SuHx) exposure on rat physiologic parameters

	Control (*n* = 30)	SuHx (*n* = 23)
BW (g)	423 ± 7	350 ± 8^a^
RV (g)	0.20 ± 0.01	0.50 ± 0.02^a^
RV/BW (mg/g)	0.48 ± 0.02	1.28 ± 0.06^a^
LV+S (g)	0.92 ± 0.02	0.95 ± 0.03
LV+S/BW (mg/g)	2.19 ± 0.04	2.79 ± 0.08^a^
HR (min^−1^)	398 ± 11	369 ± 9^a^
Hct (%)	42.5 ± 0.5	41.7 ± 1.3

BW, body weight; RV, right ventricle; LV, left ventricle; S, septum; HR, heart rate; Hct, hematocrit.

a Significant difference between control and SuHx values (*P *<* *0.05 by unpaired *t* test).

### Pulmonary vascular remodeling in SuHx

**Figure 2:**
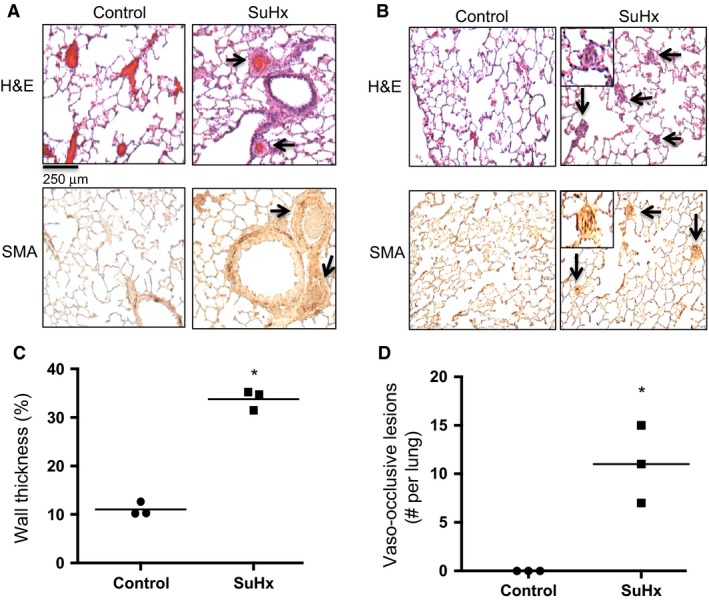
Effect of SU5416‐hypoxia (SuHx) exposure on remodeling of the rat pulmonary vasculature. Representative images show formalin‐fixed, paraffin‐embedded, sectioned rat lungs from control and SuHx rats, stained with either hematoxylin and eosin (H&E; top) or smooth muscle‐specific *α*‐actin (SMA; bottom). Arrows point to (A) vessels with thickened medial layer and (B) to vaso‐occlusive lesions. (C) Plot shows quantitative analysis of medial thickening in control and SuHx rats (*n *=* *3 each). (D) Plot shows analysis of vaso‐occlusive lesions in control and SuHx rats (*n *=* *3 each). Lines indicates mean values. *indicates significant difference between control and SuHx values (*P *<* *0.05 by unpaired *t* test).

To verify the presence of vascular remodeling as a component of PH in SuHx rats, immunohistochemistry was performed on lung sections from SuHx and normoxic control rats. Thickness of the vascular media was increased in SuHx rats (Fig. 2A and C). Additionally, vaso‐occlusive lesions were present in SuHx rats, but were not in normoxic controls (Fig. 2B and D). Thus, vascular remodeling, including medial thickening and development of vaso‐occlusive lesions, occurred in SuHx rats.

### NHE and resting pH_i_ in rat PASMCs

**Figure 3:**
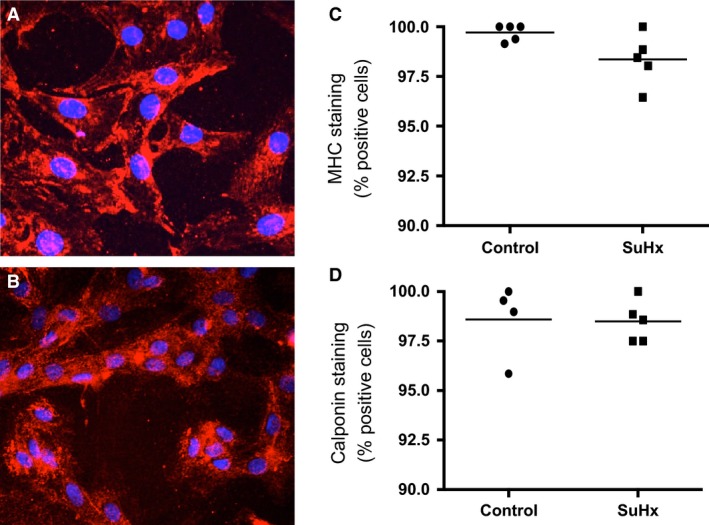
Cells isolated from rats stain positive for pulmonary arterial smooth muscle cells markers. Representative images of isolated cells stained for (A) myosin heavy chain (MHC) and (B) calponin. Both samples also had DAPI nuclear counterstain (blue). Plots show percent of DAPI‐positive cells which also stained positive for (C) MHC or (D) calponin in control (*n *=* *4–5) and SU5416‐hypoxia (*n *=* *5) rats. Lines indicate mean values.

**Figure 4:**
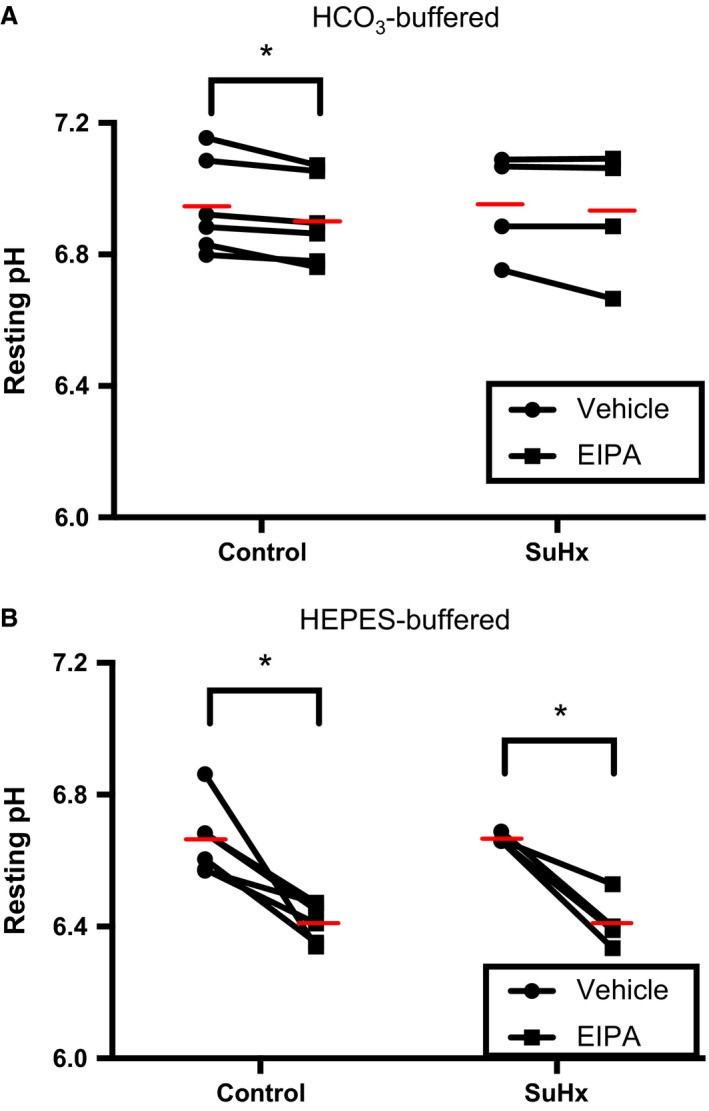
Effects of SU5416‐hypoxia (SuHx) exposure and ethyl‐isopropyl amiloride (EIPA) treatment on rat pulmonary arterial smooth muscle cells (PASMC) resting pH_i_. Plots show resting pH_i_ of PASMCs isolated from control (*n *=* *6) and SuHx (*n *=* *4) rats in the presence of 10 *μ*mol/L EIPA versus vehicle in (A) bicarbonate‐containing solution and (B) bicarbonate‐free (HEPES) solution. Red lines indicate mean values. *indicates significant difference between groups (*P *<* *0.05 by paired two‐way ANOVA with Sidak test for multiple comparisons).

We next wanted to assess whether pH_i_ homeostasis was altered in PASMCs from SuHx rats. Initial experiments were performed to verify that the cells isolated were in fact PASMCs. Using immunohistochemistry, we found that >95% of our cells exhibited positive staining for the SMC markers MHC and calponin (Fig. 3). To assess whether NHE activity is important for maintenance of resting pH_i_ of rat PASMCs in physiologic solutions, pH_i_ was measured in Krebs and HEPES1 solutions in the presence or absence of EIPA. Of note, under basal conditions, SuHx exposure did not alter resting PASMC pH_i_ in either solution (Fig. 4). In bicarbonate‐containing solution (in which bicarbonate exchangers are operative), NHE inhibition had a small effect on resting pH_i_ in control PASMCs, lowering pH_i_ by 0.04 ± 0.01 units, but had no significant effect in SuHx PASMCs, lowering resting pH_i_ by 0.02 ± 0.02 units (Fig. 4 **A**). In contrast, in bicarbonate‐free solution, EIPA had a much larger effect, lowering resting pH_i_ by 0.25 ± 0.05 units in control and by 0.25 ± 0.06 units in SuHx PASMCs (Fig. 4B). These results indicate that NHE provides a significant contribution to resting pH_i_ in rat PASMCs in the absence of bicarbonate, but that the role of NHE is lessened when bicarbonate exchangers are active.

### NHE activity in SuHx PASMCs

**Figure 5:**
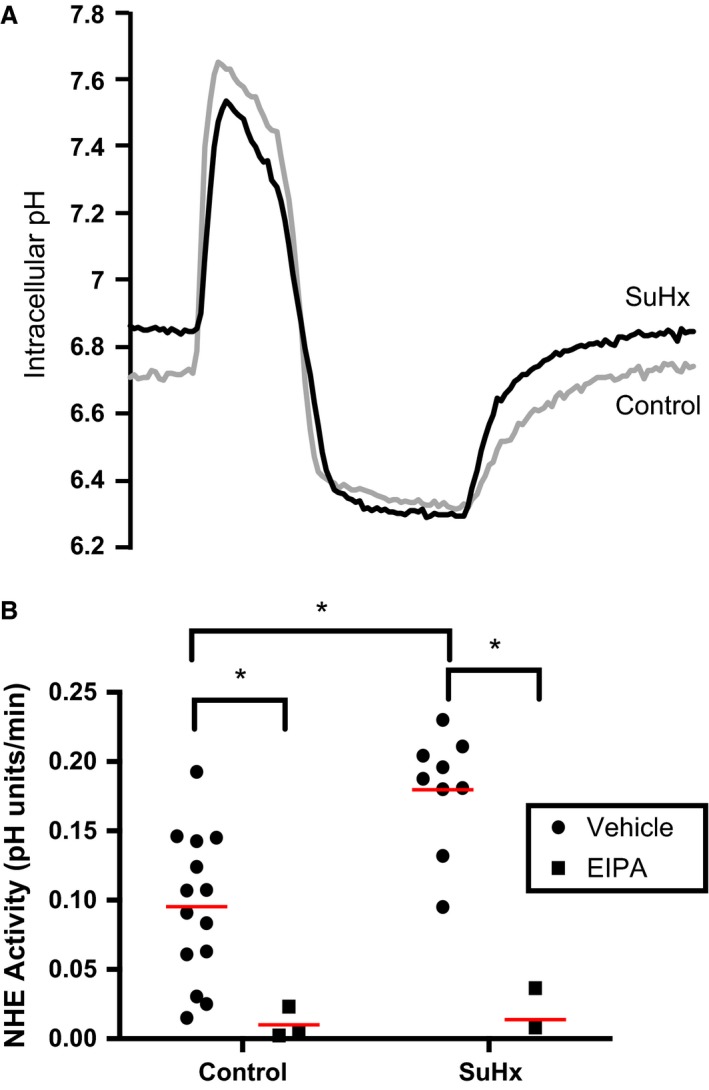
Effects of SU5416‐hypoxia (SuHx) exposure and ethyl‐isopropyl amiloride (EIPA) treatment on Na^+^/H^+^ exchanger (NHE) activity. (A) Representative traces show changes in pH_i_ in control and SuHx pulmonary arterial smooth muscle cells (PASMC)s during the ammonium pulse protocol. (B) Plots show NHE activity in PASMCs isolated from control (*n *=* *14) and SuHx (*n *=* *9) rats, as well as PASMCs treated with 10 *μ*mol/L EIPA (*n *=* *3 each). Red lines indicate mean values. *indicates significant difference between groups (*P *<* *0.05 by unpaired two‐way ANOVA with Sidak test for multiple comparisons).

To assess whether NHE activity differs in control and SuHx PASMCs, the rate of pH_i_ recovery from acid load was measured in bicarbonate‐free solution. NHE activity in PASMCs from SuHx rats (0.180 ± 0.014 pH units/min, *n* = 9) was significantly elevated compared to controls (0.095 ± 0.014 pH units/min, *n* = 14) (Fig. 5). Application of 10 *μ*mol/L EIPA decreased NHE activity to near zero in both SuHx and control PASMCs. These results indicate that NHE activity is increased in SuHx PASMCs and that EIPA successfully blocks NHE activity in cells from both control and SuHx rats.

### NHE and migration and proliferation in SuHx PASMCs

**Figure 6:**
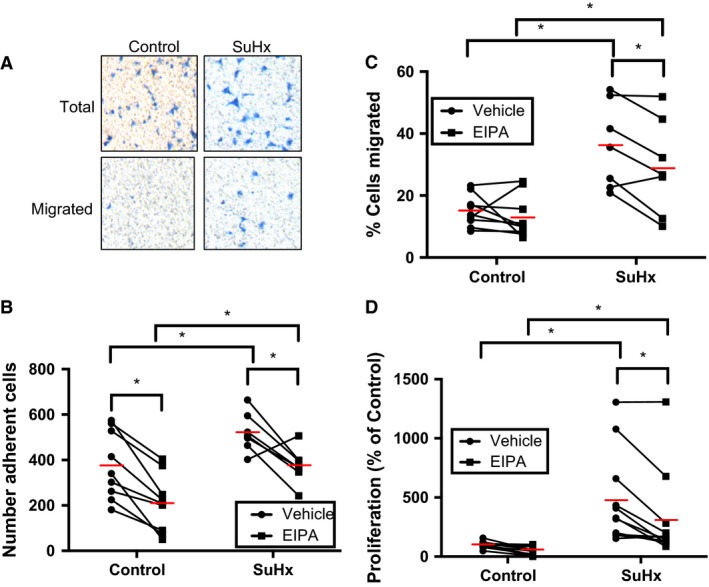
Effect of Na^+^/H^+^ exchanger (NHE) inhibition on rat pulmonary arterial smooth muscle cells (PASMC) migration and proliferation. (A) Representative images show PASMCs (stained with Brilliant Blue) from control and SU5416‐hypoxia (SuHx) rats before and after removal of unmigrated cells. Plots show effect of 10 *μ*mol/L ethyl‐isopropyl amiloride (EIPA) versus vehicle on (B) transwell filter adherence and (C) migration of PASMCs isolated from control (*n *=* *9) and SuHx (*n *=* *7) rats. (D) Plot shows effect of 10 *μ*mol/L EIPA versus vehicle on proliferation of PASMCs isolated from control (*n *=* *12) and SuHx (*n *=* *11) rats. Red lines indicate mean values. *indicates significant difference between groups (*P *<* *0.05 by paired two‐way ANOVA with Sidak test for multiple comparisons).

In order to gauge the role NHE plays in PASMC function in normal and SuHx rats, PASMC migration and proliferation were measured in the presence of EIPA or vehicle. Using total number of cells on the membrane as a measure of adherence, our data suggest that PASMCs from SuHx rats were more adherent to the filter (522 ± 32 cells, *n* = 7) than PASMCs from control rats (376 ± 50 cells, *n* = 9; Fig. 6B). Migration of PASMCs from SuHx rats (36 ± 5% cells migrated, *n* = 7) was increased compared to controls (15 ± 2% cells migrated, *n* = 9; Fig. 6C). EIPA exposure decreased the number of adherent PASMCs from both SuHx (375 ± 30 cells, *n* = 7) and control (208 ± 42 cells, *n* = 9) rats, with no significant interaction between the SuHx and EIPA variables upon adherence. Furthermore, EIPA treatment significantly decreased migration in PASMCs from SuHx rats (29 ± 6% cells migrated, *n* = 7) but not control rats (13 ± 2% cells migrated, *n* = 9). Although the decrease in migration between vehicle‐ and EIPA‐treated PASMCs from control rats did not reach significance, the interaction between the SuHx and EIPA variables upon migration also did not reach significance (*P *=* *0.17) due to substantial intragroup variability. Thus, we cannot conclude that the effect of NHE inhibition upon migration is specific to SuHx PASMCs.

With respect to cell growth, proliferation of PASMCs from SuHx rats (477 ± 116%, *n* = 11) was significantly increased compared to controls (100 ± 7%, *n* = 12; Fig. 6D). Similar to the result obtain in migration experiments, EIPA decreased the proliferation of SuHx PASMCs (309 ± 112%, *n* = 11) but did not significantly affect control PASMCs (53 ± 11%, *n* = 12). However, in this case, there was significant difference in the effect of NHE inhibition between SuHx and control PASMCs (*P *<* *0.05 for interaction).

Taken together, these results indicate that PASMC migration and proliferation are increased by the SuHx exposure and decreased by NHE inhibition.

### NHE1 protein expression in PASMCs from SuHx rats

**Figure 7:**
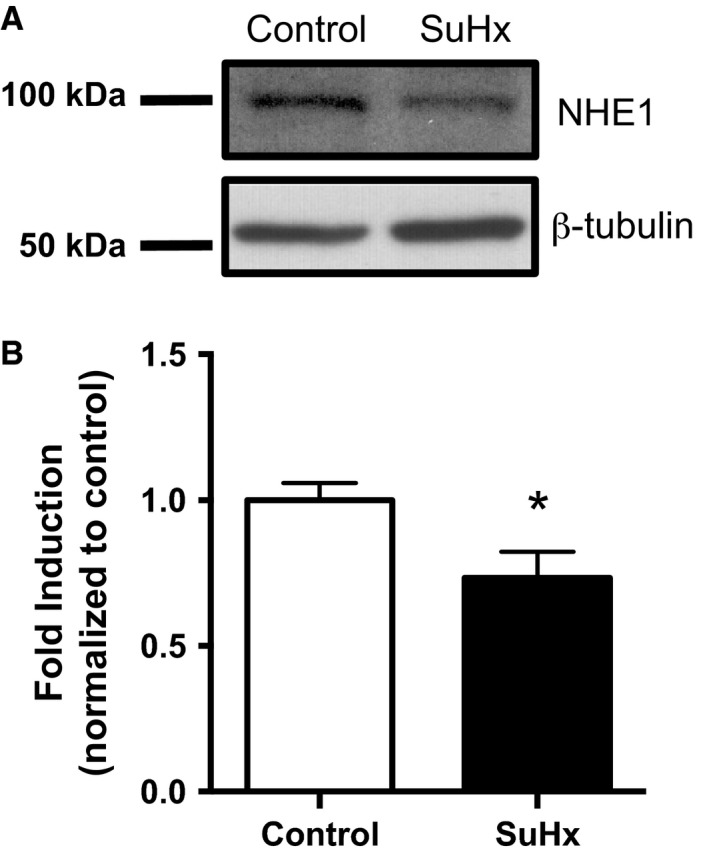
Effect of SU5416‐hypoxia (SuHx) exposure on Na^+^/H^+^ exchanger (NHE)1 protein expression in rat pulmonary arterial smooth muscle cells (PASMC)s. (A) Representative immunoblot shows decreased NHE1 protein expression in PASMCs from SuHx rats. Exposure to SuHx had no effect on protein levels of *β*‐tubulin loading controls. (B) Bar graph shows mean fold induction in NHE1 protein expression normalized to *β*‐tubulin expression (ratio of NHE1/*β*‐tubulin in control rats set to 1) in PASMCs isolated from control (*n *=* *9) and SuHx (*n *=* *8) rats. Whiskers indicate standard errors. *indicates significant difference between control and SuHx values (*P *<* *0.05 by unpaired *t* test).

Previous reports indicate that increased NHE activity in PASMCs from animals with hypoxia‐induced PH was associated with increased NHE1 mRNA and protein (Rios et al. 2005; Shimoda et al. 2006). To determine whether the same was true in SuHx, immunoblot analysis was used to evaluate NHE1 expression in PASMCs from control and SuHx rats. In contrast to previously reported results obtained with hypoxia alone, NHE1 protein expression in PASMCs isolated from SuHx rats was decreased slightly, but significantly, by 27% relative to controls (Fig. 7).

### Effect of ROCK upon NHE activity in PASMCs

**Figure 8:**
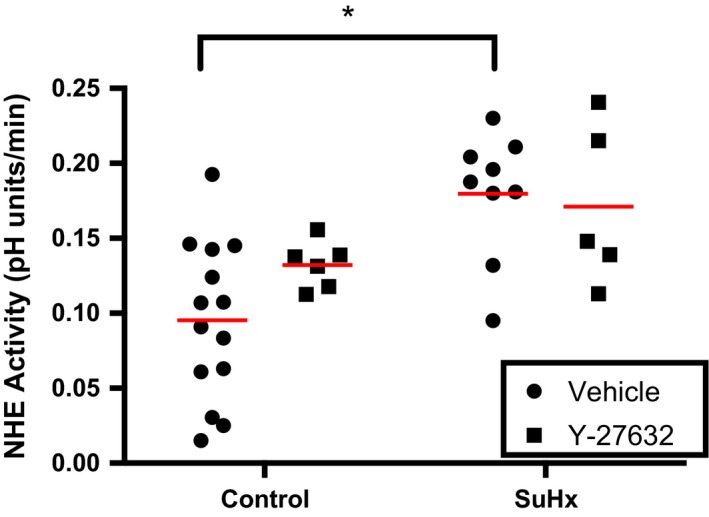
Effect of rho kinase inhibition on Na^+^/H^+^ exchanger (NHE) activity. Plot shows NHE activity in pulmonary arterial smooth muscle cells (PASMC)s isolated from control (*n *=* *14) and SU5416‐hypoxia (SuHx) (*n *=* *9) rats, as well as NHE activity in control (*n *=* *6) and SuHx (*n *=* *5) PASMCs treated with 10 *μ*mol/L Y‐27632. Red lines indicate mean values. * indicates significant difference between groups (*P *<* *0.05 by unpaired two‐way ANOVA with Sidak test for multiple comparisons).

Given that elevated NHE activity in SuHx PASMCs was not associated with increased NHE1 protein expression, we sought to determine other pathways that could account for the increased NHE activity. Acute reduction in pulmonary arterial pressure in SuHx rats treated with ROCK inhibitor suggests that ROCK is activated in this model (Oka et al. 2007) and prior work indicates that ROCK signaling mediates enhanced PASMC NHE activity in response to endothelin‐1 (ET‐1) stimulation (Undem et al. 2012). Thus, we tested the effect of the ROCK inhibitor, Y‐27632, on NHE activity. In PASMCs treated with Y‐27632 (10 *μ*mol/L), we found that ROCK inhibition had no significant effect on NHE activity in control PASMCs (Fig. 8). Pharmacologic ROCK inhibition also had no effect on NHE activity in PASMCs from SuHx rats (0.180 ± 0.014 pH units/min, *n* = 9 for SuHx vs. 0.171 ± 0.024 pH units/min, *n* = 5 for SuHx +Y‐27632). These results indicate that the increase in NHE activity in SuHx PASMCs is not mediated by ROCK activation.

## Discussion

Previous work demonstrated a critical role for increased NHE1 expression and activity in mediating PASMC migration and proliferation in the hypoxia‐induced model of PH. However, the role of NHE in forms of PH not associated with persistent hypoxia, such as PAH, remained undetermined. In this study, we found that NHE activity in PASMCs isolated from SuHx rats, a model of severe PH reminiscent of human PAH, was increased compared to controls. Moreover, PASMCs from SuHx rats exhibited enhanced migratory and proliferative capacity ex vivo, which was reduced when NHE was inhibited. However, unlike models where hypoxia was used to induce PH, the increase in NHE activity was not correlated with either an alkaline shift in resting pH_i_ or elevated NHE1 expression. These data provide evidence that increased NHE activity represents an important common pathway that is necessary for pathologic PASMC proliferation and migration in varied groups of PH. Furthermore, our findings of NHE‐dependent increases in PASMC proliferation and migration, despite unchanged pH_i_, suggest that the effects of NHE on PASMC function may not be due simply to alterations in pH_i_ homeostasis.

The SuHx rat model of PH is superior to the CH model in its recapitulation of features of human PAH in several ways: SuHx rats develop more severe elevations in pulmonary arterial pressures (Taraseviciene‐Stewart et al. 2001), form vaso‐occlusive lesions (Taraseviciene‐Stewart et al. 2001), and exhibit progressive pulmonary vascular remodeling after return to normoxia (Abe et al. 2010). Our findings of substantial medial thickening as well as formation of vaso‐occlusive lesions in SuHx rats are similar to prior reports (Taraseviciene‐Stewart et al. 2001). Hct, which is elevated by hypoxia, returned to normal by completion of the SuHx exposure. Of note, LV weight (normalized to body weight) was mildly increased in SuHx rats and heart rate was slightly decreased, which is a finding that has been noted previously in SuHx rats (Abe et al. 2010). While the mechanisms underlying altered LV mass and heart rate remain unclear, these results suggest that the effects of SuHx may not be limited to the pulmonary circulation. Nonetheless, SuHx exposure in our rats led to significant remodeling of the pulmonary vasculature. Interestingly, in the SuHx model (and potentially in human PAH as well), medial thickening precedes intimal remodeling and formation of occlusive lesions, suggesting that pathologic changes in PASMC function may represent the primary defect (Tuder 2014).

NHE has been shown to regulate the resting pH_i_ of PASMCs from both mice (Rios et al. 2005) and guinea pigs (Quinn et al. 1991), but was found to not contribute significantly to the resting pH_i_ of cat PASMCs (Madden et al. 2001). Our findings that NHE inhibition decreased the resting pH_i_ of rat PASMCs in bicarbonate‐free, and to a lesser extent in bicarbonate‐containing solution, indicate that Na^+^/H^+^ exchange contributes to pH_i_ homeostasis in the rat as well. That the contribution of NHE to resting pH_i_ was clearly less in the presence of bicarbonate suggests that bicarbonate exchangers can compensate to maintain pH_i_ homeostasis when NHE is inactivated. The resting pH_i_ values in bicarbonate‐free conditions that we measured in rat PASMCs are consistent with prior measurements from our laboratory (Shimoda et al. 2006), and while relatively lower than the resting pH_i_ measured previously in mouse (Rios et al. 2005) or guinea pig (Quinn et al. 1991) PASMCs, are similar to values measured in cat PASMCs (Madden et al. 2001). The exact reason for these differences between reported basal pH_i_ are currently unknown, but could represent differences between organisms, culture conditions, and/or differences in calibration methods of pH_i_ measurements.

Surprisingly, resting pH_i_ was unaltered in PASMCs from SuHx rats in both bicarbonate‐containing and bicarbonate‐free solutions, despite increased NHE activity. This finding is in stark contrast to the NHE‐mediated alkaline shift in pH_i_ observed with hypoxia (Rios et al. 2005; Shimoda et al. 2006). It should be noted, however, that while PASMCs from the SuHx rat are exposed in vivo to 3 weeks of 10% O_2_, the hypoxic exposure is then followed by 2 weeks of in vivo return to normoxia as well as several days of culture under nonhypoxic conditions. Thus, it is possible that the increase in resting pH_i_ observed previously is a direct result of hypoxia, perhaps requiring the upregulation of NHE and altered expression/activity of an additional, as yet unknown, factor. In the SuHx model, prolonged removal from the hypoxic environment could result in the return of this “factor,” and consequently pH_i_, to normal levels. Another possible explanation for this discrepancy is that intracellular buffering mechanisms may compensate for increased NHE activity in SuHx PASMCs at physiologic pH_i_, such that no change in resting pH_i_ is evident despite increased NHE activity.

To our knowledge, NHE activity has not been measured in PASMCs from a model of PAH. Our findings show PASMC NHE activity in the SuHx rat is elevated, and that the increase is similar in magnitude to that observed in rat PASMCs following exposure to ex vivo hypoxia (Shimoda et al. 2006) and in mouse PASMCs following in vivo exposure to CH (Rios et al. 2005). In stark contrast to the results observed in CH (Rios et al. 2005; Shimoda et al. 2006), NHE1 expression was not increased in SuHx PASMCs and thus does not account for the increase in NHE activity. Since our experimental conditions resulted in similar pH gradients during the ammonium pulse protocol, a difference in driving H^+^ ion gradient also cannot account for the difference in activity. While our experiments could not determine the mechanism underlying the increased PASMC NHE activity in the SuHx rat, a potential possibility could be via phosphorylation of the NHE1 C‐terminal tail, which has been shown to activate NHE in response to a wide array of mitogens (Sardet et al. 1990) and is mediated by a range of different kinases in various cell types (Touyz and Schiffrin 1997; Moor and Fliegel 1999; Maekawa et al. 2006; Vila‐Petroff et al. 2010; Undem et al. 2012). In particular, our laboratory has shown that ET‐1, a circulating factor know to be upregulated in the serum of PH patients (Rubens et al. 2001), increased NHE activity in normoxic rat PASMCs via activation of ROCK (Undem et al. 2012). Furthermore, increased ROCK activity has been noted in SuHx rat lungs compared to normoxic controls (Oka et al. 2007). We found, however, that increased NHE activity in SuHx PASMCs was not altered when ROCK was inhibited. It is unlikely that the inability of ROCK inhibition to reduce NHE activity was due to incomplete inhibition of the kinase, as we have previously demonstrated that the concentration of drug used in the current experiments was sufficient to completely prevent ET‐1‐induced increases in NHE activity (Undem et al. 2012).

While our results appear to rule out ROCK as a mediator of increased NHE activity in SuHx PASMCs, there are other kinases that also have been shown to regulate NHE activity. For example, c‐Src and extracellular signal‐regulated kinase signaling mediate acid‐induced activation of NHE3 in renal tubular epithelial cell lines (Tsuganezawa et al. 2002). This suggests the possibility that, at physiologic pH_i_, NHE activity is similar in control and SuHx PASMCs (and, thus, resting pH_i_ is equivalent) but that acid load uniquely activates NHE in SuHx cells through upregulated kinase signaling. Thus, future experiments will be needed to determine whether phosphorylation by kinases other than ROCK and/or enhanced sensitivity to acid loading are responsible for increased NHE activity observed in the SuHx rat model of PAH and, if so, which kinases are involved.

Previous studies have clearly demonstrated that exposure to hypoxia is sufficient to induce NHE1 expression and increase NHE activity in PASMCs and that this hypoxic upregulation of NHE1 is mediated by HIF‐1 (Rios et al. 2005; Shimoda et al. 2006). While acid‐induced NHE activity was increased in PASMCs from SuHx rats, our immunoblot results suggest that this was accomplished via a different mechanism than in response to hypoxia, where enhanced NHE activity was associated with a HIF‐dependent increase in NHE1 protein expression (Rios et al. 2005; Shimoda et al. 2006). Indeed, to our surprise, NHE1 protein expression in PASMCs was slightly decreased in SuHx rats compared to control rats. It is possible that the lack of increased NHE1 protein expression following the SuHx exposure may be due to loss of HIF‐1 activation during the 2‐week return to normoxia at the end of the SuHx protocol. We focused on the expression of NHE1 because we have found that NHE1 is expressed in rat PASMCs, while NHE2 and NHE3 are not (Shimoda et al. 2006). NHE3–5 expression is absent in whole lung tissue preparations and NHE6–10 are known to have more restricted tissue and/or organellar‐specific localization (Huetsch and Shimoda 2015). These patterns of expression indicate that NHE1 is the primary plasma membrane isoform controlling cytosolic pH_i_ in PASMCs. Nonetheless, we cannot rule out that expression of a different isoform was altered and is responsible for increased NHE activity in SuHx.

Our novel findings of increased migratory and proliferative capacity of PASMCs isolated from SuHx rats provide important evidence further validating that this animal model reproduces key features of human PAH. PASMC proliferation (Falcetti et al. 2010) and migration (Paulin et al. 2014) are both increased in human PAH PASMCs and are important contributors to the vascular remodeling process. To our knowledge, increased PASMC migration in the SuHx rat model has not been previously reported and while other in vivo studies showed via immunohistochemistry that proliferative markers (i.e., PCNA and Ki67) are increased in SuHx rat PASMCs (Taraseviciene‐Stewart et al. 2001; Meloche et al. 2014), our results demonstrate that SuHx PASMCs retain this proliferative phenotype in culture. Not only does this finding allow for ex vivo assays of PASMC function, but it also indicates that the enhanced proliferative capacity of these cells does not require exposure to circulating or paracrine factors, or hemodynamic stress. It is important to note that while our results show that NHE inhibition reduced PASMC migration and proliferation in vitro, we have not yet tested the effect of NHE inhibition in vivo. However, inhibition of NHE decreased hypoxia‐induced PASMC migration and proliferation in vitro (Yu and Hales 2011) and prevented hypoxia‐induced vascular remodeling in vivo (Quinn et al. 1998; Yu et al. 2008), suggesting that effects of NHE on PASMC function in vitro appear to correlate with its effects upon vascular remodeling in vivo.

In our migration assays, the total number of PASMCs adherent to the transwell filter appeared to be greater in SuHx rats than in controls, whereas EIPA reduced the number of adherent cells, in addition to decreasing migration rates. While it is certainly possible that the SuHx exposure and EIPA treatment directly affect the ability of PASMCs to adhere to the filter surface, we consider it a more likely possibility that the effects of SuHx exposure and EIPA on PASMC proliferation are reflected in the differences in the total number of cells adherent to the filter. Keeping this important consideration in mind, we measured migration as the percent of cells traversing the filter, rather than the raw number of migrated cells (i.e., cells on the bottom of the filter), which should mitigate any effect of differences in total cell number due to proliferation.

NHE inhibition has been shown previously to attenuate hypoxia‐induced enhancement of PASMC proliferation and migration (Yu and Hales 2011). Our findings that EIPA decreased both proliferation and migration in PASMCs from SuHx rats indicate that NHE activity is also important to pathologic PASMC function in PAH. Interestingly, the effect of NHE inhibition on migration was not specific to SuHx PASMCs. This result suggests that while NHE activity is necessary for migration in PASMCs, it may not be the driving factor for the enhanced migration observed in SuHx. Nonetheless, the finding that EIPA inhibits migration, while not specific to SuHx PASMCs, remains interesting as it suggests that this drug could play a beneficial role in inhibiting vascular remodeling in PAH, with the important caveat that it could also have a significant impact upon migration of normal cells as well.

The exact mechanism by which NHE activity facilitates proliferation and migration in PASMCs remains unresolved. EIPA clearly prevented NHE ion exchange activity but, given that SuHx PASMCs were not alkalinized relative to controls, it is unlikely that the changes in PASMC function in SuHx are due to altered pH_i_ homeostasis or that the effect of EIPA was mediated by reductions in NHE ion exchange. Interestingly, NHE is known to have effects on cell behavior independent of its ion exchange functionality. Specifically, fibroblast migration was impaired in the setting of mutations to the NHE1 C‐terminal tail, which disrupted interactions between NHE1 and the cytoskeleton (Denker and Barber 2002). Whether EIPA exerts an effect upon the NHE1 C‐terminus or NHE1 interactions with the cytoskeleton remains undefined but this possibility offers a potential mechanism for NHE‐dependent alterations in cell function in the absence of modified pH_i_ homeostasis.

It should be noted that although EIPA is most potent against NHE1, it also inhibits other NHE isoforms (Masereel 2003). As mentioned earlier, we found that NHE1 is the primary isoform controlling cytosolic pH_i_ in PASMCs. While EIPA does not exert a specific inhibitory effect upon NHE1, the known isoform expression patterns suggest that the effect of EIPA upon PASMC function is likely due to inhibition of NHE1.

In summary, the results of the current study demonstrate that NHE inhibition attenuates increased PASMC migration and proliferation in the SuHx rat model of PAH. While NHE has been shown to be necessary for the development of vascular remodeling in response to CH in the mouse, our findings suggest that NHE likely contributes to vascular remodeling in a rat model of severe PH that resembles PAH and thus may represent a common pathway leading to vascular remodeling in different groups of PH. Since PASMCs from the SuHx model exhibit enhanced migratory and proliferative capacity without alterations in resting pH_i_, our results also indicate that the effects of NHE on PASMC function are not mediated by changes in pH_i_ homeostasis. Further study will be needed to dissect the exact mechanism whereby NHE inhibition alters PASMC function as well as the ability of NHE inhibition to prevent and/or reverse remodeling in the SuHx model in vivo.

## Conflict of Interest

None declared.

## References

[phy212729-bib-0001] Abe, K. , M. Toba , A. Alzoubi , M. Ito , K. A. Fagan , C. D. Cool , et al. 2010 Formation of plexiform lesions in experimental severe pulmonary arterial hypertension. Circulation 121:2747–2754. 20547927 10.1161/CIRCULATIONAHA.109.927681

[phy212729-bib-0002] Attaphitaya, S. , K. Park , and J. E. Melvin . 1999 Molecular cloning and functional expression of a rat Na+/H+ exchanger (NHE5) highly expressed in brain. J. Biol. Chem. 274:4383–4388. 9933642 10.1074/jbc.274.7.4383

[phy212729-bib-0003] Brant, S. R. , C. H. Yun , M. Donowitz , and C. M. Tse . 1995 Cloning, tissue distribution, and functional analysis of the human Na+/N+ exchanger isoform, NHE3. Am. J. Physiol. 269:C198–206. 7631746 10.1152/ajpcell.1995.269.1.C198

[phy212729-bib-0004] Chazova, I. , J. E. Loyd , V. S. Zhdanov , J. H. Newman , Y. Belenkov , and B. Meyrick . 1995 Pulmonary artery adventitial changes and venous involvement in primary pulmonary hypertension. Am. J. Pathol. 146:389–397. 7856750 PMC1869854

[phy212729-bib-0005] Denker, S. P. , and D. L. Barber . 2002 Cell migration requires both ion translocation and cytoskeletal anchoring by the Na‐H exchanger NHE1. J. Cell Biol. 159:1087–1096. 12486114 10.1083/jcb.200208050 PMC2173980

[phy212729-bib-0006] Falcetti, E. , S. M. Hall , P. G. Phillips , J. Patel , N. W. Morrell , S. G. Haworth , et al. 2010 Smooth muscle proliferation and role of the prostacyclin (IP) receptor in idiopathic pulmonary arterial hypertension. Am. J. Respir. Crit. Care Med. 182:1161–1170. 20622039 10.1164/rccm.201001-0011OC PMC3001258

[phy212729-bib-0007] Farrukh, I. S. , J. R. Barry , and W. H. Barry . 1996 Effect of intracellular pH on ferret pulmonary arterial smooth muscle cell calcium homeostasis and pressure. J. Appl. Physiol. (1985) 80:496–505. 8929590 10.1152/jappl.1996.80.2.496

[phy212729-bib-0008] Huetsch, J. , and L. A. Shimoda . 2015 Na(+)/H(+) exchange and hypoxic pulmonary hypertension. Pulm. Circ. 5:228–243. 26064449 10.1086/680213 PMC4449235

[phy212729-bib-0009] Ling, Y. , M. K. Johnson , D. G. Kiely , R. Condliffe , C. A. Elliot , J. S. Gibbs , et al. 2012 Changing demographics, epidemiology, and survival of incident pulmonary arterial hypertension: results from the pulmonary hypertension registry of the United Kingdom and Ireland. Am. J. Respir. Crit. Care Med. 186:790–796. 22798320 10.1164/rccm.201203-0383OC

[phy212729-bib-0010] Madden, J. A. , D. E. Ray , P. A. Keller , and J. G. Kleinman . 2001 Ion exchange activity in pulmonary artery smooth muscle cells: the response to hypoxia. Am. J. Physiol. Lung Cell. Mol. Physiol. 280:L264–271. 11159005 10.1152/ajplung.2001.280.2.L264

[phy212729-bib-0011] Maekawa, N. , J. Abe , T. Shishido , S. Itoh , B. Ding , V. K. Sharma , et al. 2006 Inhibiting p90 ribosomal S6 kinase prevents (Na+)‐H+ exchanger‐mediated cardiac ischemia‐reperfusion injury. Circulation 113:2516–2523. 16717153 10.1161/CIRCULATIONAHA.105.563486

[phy212729-bib-0012] Masereel, B. 2003 An overview of inhibitors of Na+/H+ exchanger. Eur. J. Med. Chem. 38:547–554. 12832126 10.1016/s0223-5234(03)00100-4

[phy212729-bib-0013] Meloche, J. , A. Pflieger , M. Vaillancourt , R. Paulin , F. Potus , S. Zervopoulos , et al. 2014 Role for DNA damage signaling in pulmonary arterial hypertension. Circulation 129:786–797. 24270264 10.1161/CIRCULATIONAHA.113.006167

[phy212729-bib-0014] Miyazaki, E. , M. Sakaguchi , S. Wakabayashi , M. Shigekawa , and K. Mihara . 2001 NHE6 protein possesses a signal peptide destined for endoplasmic reticulum membrane and localizes in secretory organelles of the cell. J. Biol. Chem. 276:49221–49227. 11641397 10.1074/jbc.M106267200

[phy212729-bib-0015] Moor, A. N. , and L. Fliegel . 1999 Protein kinase‐mediated regulation of the Na(+)/H(+) exchanger in the rat myocardium by mitogen‐activated protein kinase‐dependent pathways. J. Biol. Chem. 274:22985–22992. 10438464 10.1074/jbc.274.33.22985

[phy212729-bib-0016] Nakamura, N. , S. Tanaka , Y. Teko , K. Mitsui , and H. Kanazawa . 2005 Four Na+/H+ exchanger isoforms are distributed to Golgi and post‐Golgi compartments and are involved in organelle pH regulation. J. Biol. Chem. 280:1561–1572. 15522866 10.1074/jbc.M410041200

[phy212729-bib-0017] Numata, M. , and J. Orlowski . 2001 Molecular cloning and characterization of a novel (Na+, K+)/H+ exchanger localized to the trans‐Golgi network. J. Biol. Chem. 276:17387–17394. 11279194 10.1074/jbc.M101319200

[phy212729-bib-0018] Numata, M. , K. Petrecca , N. Lake , and J. Orlowski . 1998 Identification of a mitochondrial Na+/H+ exchanger. J. Biol. Chem. 273:6951–6959. 9507001 10.1074/jbc.273.12.6951

[phy212729-bib-0019] Oka, M. , N. Homma , L. Taraseviciene‐Stewart , K. G. Morris , D. Kraskauskas , N. Burns , et al. 2007 Rho kinase‐mediated vasoconstriction is important in severe occlusive pulmonary arterial hypertension in rats. Circ. Res. 100:923–929. 17332430 10.1161/01.RES.0000261658.12024.18

[phy212729-bib-0020] Orlowski, J. , R. A. Kandasamy , and G. E. Shull . 1992 Molecular cloning of putative members of the Na/H exchanger gene family. cDNA cloning, deduced amino acid sequence, and mRNA tissue expression of the rat Na/H exchanger NHE‐1 and two structurally related proteins. J. Biol. Chem. 267:9331–9339. 1577762

[phy212729-bib-0021] Paulin, R. , J. Meloche , A. Courboulin , C. Lambert , A. Haromy , A. Courchesne , et al. 2014 Targeting cell motility in pulmonary arterial hypertension. Eur. Respir. J. 43:531–544. 23845719 10.1183/09031936.00181312

[phy212729-bib-0022] Quinn, D. A. , T. W. Honeyman , P. M. Joseph , B. T. Thompson , C. A. Hales , and C. R. Scheid . 1991 Contribution of Na+/H+ exchange to pH regulation in pulmonary artery smooth muscle cells. Am. J. Respir. Cell Mol. Biol. 5:586–591. 1659836 10.1165/ajrcmb/5.6.586

[phy212729-bib-0023] Quinn, D. A. , C. G. Dahlberg , J. P. Bonventre , C. R. Scheid , T. Honeyman , P. M. Joseph , et al. 1996 The role of Na+/H+ exchange and growth factors in pulmonary artery smooth muscle cell proliferation. Am. J. Respir. Cell Mol. Biol. 14:139–145. 8630263 10.1165/ajrcmb.14.2.8630263

[phy212729-bib-0024] Quinn, D. A. , H. K. Du , B. T. Thompson , and C. A. Hales . 1998 Amiloride analogs inhibit chronic hypoxic pulmonary hypertension. Am. J. Respir. Crit. Care Med. 157:1263–1268. 9563749 10.1164/ajrccm.157.4.9704106

[phy212729-bib-0025] Rios, E. J. , M. Fallon , J. Wang , and L. A. Shimoda . 2005 Chronic hypoxia elevates intracellular pH and activates Na+/H+ exchange in pulmonary arterial smooth muscle cells. Am. J. Physiol. Lung Cell. Mol. Physiol. 289:L867–874. 15964895 10.1152/ajplung.00455.2004

[phy212729-bib-0026] Rubens, C. , R. Ewert , M. Halank , R. Wensel , H. D. Orzechowski , H. P. Schultheiss , et al. 2001 Big endothelin‐1 and endothelin‐1 plasma levels are correlated with the severity of primary pulmonary hypertension. Chest 120:1562–1569. 11713135 10.1378/chest.120.5.1562

[phy212729-bib-0027] Sardet, C. , L. Counillon , A. Franchi , and J. Pouyssegur . 1990 Growth factors induce phosphorylation of the Na+/H+ antiporter, glycoprotein of 110 kD. Science 247:723–726. 2154036 10.1126/science.2154036

[phy212729-bib-0028] Shimoda, L. A. , and S. S. Laurie . 2013 Vascular remodeling in pulmonary hypertension. J. Mol. Med. (Berl) 91:297–309. 23334338 10.1007/s00109-013-0998-0 PMC3584237

[phy212729-bib-0029] Shimoda, L. A. , M. Fallon , S. Pisarcik , J. Wang , and G. L. Semenza . 2006 HIF‐1 regulates hypoxic induction of NHE1 expression and alkalinization of intracellular pH in pulmonary arterial myocytes. Am. J. Physiol. Lung Cell. Mol. Physiol. 291:L941–949. 16766575 10.1152/ajplung.00528.2005

[phy212729-bib-0030] Simonneau, G. , I. M. Robbins , M. Beghetti , R. N. Channick , M. Delcroix , C. P. Denton , et al. 2009 Updated clinical classification of pulmonary hypertension. J. Am. Coll. Cardiol. 54:S43–54. 19555858 10.1016/j.jacc.2009.04.012

[phy212729-bib-0031] Taraseviciene‐Stewart, L. , Y. Kasahara , L. Alger , P. Hirth , G. MC Mahon , J. Waltenberger , et al. 2001 Inhibition of the VEGF receptor 2 combined with chronic hypoxia causes cell death‐dependent pulmonary endothelial cell proliferation and severe pulmonary hypertension. FASEB J. 15:427–438. 11156958 10.1096/fj.00-0343com

[phy212729-bib-0032] Touyz, R. M. , and E. L. Schiffrin . 1997 Growth factors mediate intracellular signaling in vascular smooth muscle cells through protein kinase C‐linked pathways. Hypertension 30:1440–1447. 9403565 10.1161/01.hyp.30.6.1440

[phy212729-bib-0033] Tsuganezawa, H. , S. Sato , Y. Yamaji , P. A. Preisig , O. W. Moe , and R. J. Alpern . 2002 Role of c‐SRC and ERK in acid‐induced activation of NHE3. Kidney Int. 62:41–50. 12081562 10.1046/j.1523-1755.2002.00418.x

[phy212729-bib-0034] Tuder, R. M. 2009 Pathology of pulmonary arterial hypertension. Semin. Respir. Crit. Care Med. 30:376–385. 19634077 10.1055/s-0029-1233307

[phy212729-bib-0035] Tuder, R. M. 2014 How do we measure pathology in PAH (lung and RV) and what does it tell us about the disease. Drug Discov. Today 19:1257–1263. 24881780 10.1016/j.drudis.2014.05.022

[phy212729-bib-0036] Undem, C. , E. J. Rios , J. Maylor , and L. A. Shimoda . 2012 Endothelin‐1 augments Na(+)/H(+) exchange activity in murine pulmonary arterial smooth muscle cells via Rho kinase. PLoS One 7:e46303. 23029469 10.1371/journal.pone.0046303 PMC3460862

[phy212729-bib-0037] Vila‐Petroff, M. , C. Mundina‐Weilenmann , N. Lezcano , A. K. Snabaitis , M. A. Huergo , C. A. Valverde , et al. 2010 Ca(2 + )/calmodulin‐dependent protein kinase II contributes to intracellular pH recovery from acidosis via Na(+)/H(+) exchanger activation. J. Mol. Cell. Cardiol. 49:106–112. 20026127 10.1016/j.yjmcc.2009.12.007 PMC2883686

[phy212729-bib-0038] Wang, Z. , J. Orlowski , and G. E. Shull . 1993 Primary structure and functional expression of a novel gastrointestinal isoform of the rat Na/H exchanger. J. Biol. Chem. 268:11925–11928. 7685026

[phy212729-bib-0039] Wu, D. , H. Doods , and J. M. Stassen . 2006 Inhibition of human pulmonary artery smooth muscle cell proliferation and migration by sabiporide, a new specific NHE‐1 inhibitor. J. Cardiovasc. Pharmacol. 48:34–40. 16954819 10.1097/01.fjc.0000239691.69346.6a

[phy212729-bib-0040] Yu, L. , and C. A. Hales . 2011 Silencing of sodium‐hydrogen exchanger 1 attenuates the proliferation, hypertrophy, and migration of pulmonary artery smooth muscle cells via E2F1. Am. J. Respir. Cell Mol. Biol. 45:923–930. 21454803 10.1165/rcmb.2011-0032OC PMC3262694

[phy212729-bib-0041] Yu, A. Y. , L. A. Shimoda , N. V. Iyer , D. L. Huso , X. Sun , R. McWilliams , et al. 1999 Impaired physiological responses to chronic hypoxia in mice partially deficient for hypoxia‐inducible factor 1alpha. J. Clin. Invest. 103:691–696. 10074486 10.1172/JCI5912 PMC408131

[phy212729-bib-0042] Yu, L. , D. A. Quinn , H. G. Garg , and C. A. Hales . 2008 Deficiency of the NHE1 gene prevents hypoxia‐induced pulmonary hypertension and vascular remodeling. Am. J. Respir. Crit. Care Med. 177:1276–1284. 18310478 10.1164/rccm.200710-1522OC PMC2408441

